# Ethical Handling of Occupational Health and Safety Data in the Fire Service: Empirical Interview and Focus Group Study of Firefighter and Fire Service Leadership Privacy Preferences

**DOI:** 10.2196/84465

**Published:** 2026-04-02

**Authors:** Rachel J Topazian, Aleksandra Wec, Joseph Ali, Shannon Frattaroli, Cassandra K Crifasi

**Affiliations:** 1 Division of General Internal Medicine, Center for Bioethics and Humanities, University of Colorado, 13080 E. 19th Ave, Mail Stop B137, Aurora, CO, 80045, United States, 1 303-724-6392; 2 Department of Health Policy and Management, Johns Hopkins Bloomberg School of Public Health, Johns Hopkins University, Baltimore, MD, United States; 3 Department of International Health and Berman Institute of Bioethics, Johns Hopkins Bloomberg School of Public Health, Johns Hopkins University, Baltimore, MD, United States

**Keywords:** firefighter, occupational health and safety, data privacy, mental health data, data access, data sharing

## Abstract

**Background:**

There are ongoing efforts to collect larger and higher-quality amounts of occupational health and safety data to better understand and prevent injuries and fatalities among high-risk workers, such as firefighters. Digital health systems including wearable technologies, mobile apps, or internet-based data collection platforms could collect large amounts of sensitive data, but there is little evidence on worker and employer perspectives on data privacy in the fire service.

**Objective:**

Our study examined firefighters’ and fire service leadership’s preferences regarding occupational health and safety data privacy.

**Methods:**

We conducted interviews and focus groups with career firefighters in Maryland and Virginia; interviews with union representatives and department-level leaders in each state; and interviews with national-level fire service leaders in advocacy, government, and research organizations (March to November 2023). Interviews and focus groups were audio recorded and transcribed. We analyzed transcripts using thematic analysis.

**Results:**

The sample included 31 career firefighters, 2 union leaders, 11 national leaders, and 21 department-level leaders (65 total participants from 35 interviews and 4 focus groups). We identified 4 themes: acceptability of data access, sharing, and reporting practices; data sharing and access preferences; appropriate use of firefighter data; and the need for improved communication. Leaders described firefighters’ concerns about job loss and loss of privacy. Firefighters expressed general preferences that their data be deidentified and not shared widely, and they identified mental health data as important but particularly sensitive information. Firefighters also expressed frustration about sharing data with researchers or their departments without knowing the purpose or outcomes. Both firefighters and leaders emphasized the need for enhanced communication and translation of data for firefighters.

**Conclusions:**

Fire service leaders held more concerns about the use and sharing of occupational health and safety data than firefighters, but both groups identified ways to further safeguard firefighter data and improve communication about health and safety data. Future fire service data collection should incorporate privacy protections, such as limiting the collection of identifiable information and restricting data access. Data collection should be accompanied by clear communication about the purpose of the data collection, how firefighter data will be used and accessed, and the interpretation of the results. Future digital health interventions should integrate these data privacy protections to respect firefighter preferences and contribute to acceptability and uptake.

## Introduction

Firefighters take on great personal risks to provide essential services for their communities. There are approximately 1 million firefighters in the United States who respond to over 25 million calls each year [1,2]. Approximately two-thirds of these firefighters are volunteer or unpaid, and one-third are career (“paid”) firefighters [1]. Firefighters encounter a vast array of incidents, including fires, natural disasters, and medical emergencies requiring emergency medical services [2]. US firefighters sustain high rates of injury and fatality in the line of duty: in 2022, there were over 65,000 injuries and 96 fatalities that occurred at work [3,4]. Beyond on-duty incidents, firefighting has been linked to long-term adverse health outcomes, including elevated rates of certain cancers [5,6].

There are growing calls from fire service leaders, government officials, and researchers to utilize firefighter health and safety data to better understand the hazards of firefighting and design interventions to protect future generations [7-12]. Proposed data sources include national injury or disease registries, biometric or exposure data from wearable devices, and self-reported risk factors or health outcomes from other digital health platforms, such as smartphone apps [8,13,14]. While recent data collection efforts have focused on injury and disease surveillance [8,9], researchers have identified flaws in existing data infrastructure, such as large swaths of missing occupational data in cancer and burn registries, highlighting the need for more sophisticated data reporting and data linkages [9,10,12]. Federal agencies are supporting new data platforms to enhance injury surveillance and research. In 2023, the US Fire Administration began developing the National Emergency Response Information System (NERIS), a new fire service analytics platform, and the National Institute for Occupational Safety and Health created the National Firefighter Registry for Cancer [7,15,16].

While these new data initiatives may bolster health and safety efforts, expanding the collection of or access to firefighter health and safety data raises ethical concerns [8]. Additional firefighter data might help improve health and safety, but collecting these data, particularly within fire departments, raises concerns about privacy, as firefighters might be asked or required to share sensitive information with their employers, government agencies, or researchers. The fire service’s hierarchical organization—fire departments are frequently described as paramilitary organizations—adds additional complexity around data privacy, as firefighters of lower ranks may feel they have less power or choice as to whether or not they want to share personal health information with their employers.

There is little research on data privacy in the fire service, and existing studies focus on the acceptance of new technology in fire departments. Research from our study team examined perceptions of wearable data in the fire service, concluding that while leaders were optimistic about the potential for wearable data to ensure health and safety, firefighters had concerns about privacy, autonomy, and job loss [17]. Although firefighters identified major tradeoffs associated with wearable data, firefighters and leaders agreed on the need for data protections, such as deidentifying data, limiting wearable use to specific settings, providing firefighters access to their data, and preventing punitive data use. Another study examined the implementation of a biometric earpiece that firefighters could wear at a live fire, which would provide an incident commander with data including heart rate, body temperature, calories burned, and exertion estimates [18]. The study concluded that firefighters were concerned about the impact of technology on their autonomy and that firefighters were more skeptical than department leaders. Other researchers developed a biometric privacy framework based on firefighter attitudes toward a personnel management system that would use biometric information (eg, a fingerprint) to manage personnel at a fire. This study identified concerns related to workplace accountability, data access, and employer trust [19]. Collectively, these studies suggest that firefighters may have complex views on how their health and safety data are handled in the workplace, but we lack robust evidence on their privacy preferences and whether or not these preferences are understood and honored in fire departments.

Our study examined fire service members’ and their partners’ views regarding occupational health and safety data privacy. We explored data sharing, access, and communication preferences among career firefighters, fire department leaders, and national fire service leaders. Our findings highlight opportunities to improve current data privacy practices and ensure that new occupational health and safety digital health platforms, from wearables to mobile health to web-based surveys, are ethically implemented.

## Methods

### Sample and Recruitment

We purposively recruited participants from 3 groups: career (eg, “paid”) firefighters (including union leaders), fire department–level leaders, and national-level leaders. We focused on career as opposed to volunteer firefighters because we wanted to understand views on data privacy among full-time employees. We sampled fire departments that had participated in the Firefighter Organizational Culture of Safety survey. The Firefighter Organizational Culture of Safety survey is used in fire departments to assess safety climate [20]. We recruited 4 fire departments: 2 in Maryland and 2 in Virginia. We targeted departments of varying sizes that were in different regions of each state to facilitate collecting a range of perspectives. We recruited firefighters and department leaders from within the same fire departments to allow for comparisons between management and the rank-and-file within an organization. For participants within fire departments (department leaders, union leaders, firefighters), we asked a point of contact to suggest participants in specific roles whom we then invited to participate. Department leaders and union leaders were invited to participate in interviews, while career rank-and-file firefighters were invited to participate in focus group discussions. Participants were recruited via email. We encountered data collection challenges in Maryland that limited the size of the firefighter sample (see more in the *Results* and *Discussion* sections).

We conducted interviews with leaders within fire departments, including those in overall leadership, health and safety roles, or human resources. Leadership participants included civilians and sworn members of the fire service. For the national-level leaders, we conducted purposive sampling of individuals from government, advocacy, and research organizations. We also conducted snowball sampling for both department and national leaders. We believed that firefighters might hold a range of privacy views but have less experience discussing their privacy preferences. Although some individuals might feel less comfortable sharing their views in a focus group, we chose to conduct focus groups with firefighters to allow for cross talk and the exchange of ideas on an infrequently discussed topic. We anticipated that leaders might have stronger preconceived notions of privacy norms specific to their roles and chose to conduct interviews to investigate their individual views in depth.

### Data Collection

We developed interview and focus group guides to elicit views on data privacy. We aimed to understand how participants perceived several privacy domains: data collection, data use, and data access or sharing [21]. We developed questions that examined issues within these domains in the fire service and asked all participants questions within these domains, but we tailored questions as needed based on role. The guides were reviewed and refined by the study team. We collected demographic information from all participants. Multimedia Appendix 1 shows the interview and focus group discussion topics, including the key domains and high-level questions. Regardless of the data collection mode (interview vs focus group), we asked all participants about the same domains and then asked several follow-up questions to probe more deeply into interesting lines of inquiry related to the overall topic.

We conducted focus groups in person at fire stations. For some focus groups, firefighters were on duty and in service, meaning that they were available to respond to calls. If participants received a call during the discussion, it was paused or continued with the remaining participants until they returned. Interviews were conducted over Zoom and via phone. Data were collected from March to November 2023.

Consistent with qualitative research practices [22], the interviewer or moderator wrote a memo after each focus group or interview to capture initial themes and observations. All interviews and focus groups were audio recorded and transcribed verbatim by a professional transcription service.

### Analysis

We analyzed interview and focus group transcripts using thematic analysis [23]. To develop an initial codebook, 1 team member reviewed the discussion guides, memos, and transcripts and drafted an initial set of codes capturing key concepts. A second team member reviewed the guides, memos, and transcripts and then updated the draft codebook. These 2 team members both coded a subset of interview and focus group transcripts, revised the codebook, and then independently coded the remaining transcripts. They continued to meet to discuss questions about their application of the codes and whether additional codes or refinements to the definitions were needed throughout the coding process. After completing a majority of the coding, the team members examined individual codes and groupings of codes that related to data privacy. The coding team identified themes capturing core concepts and trends in the data based on these codes and groupings of codes. We determined that we had reached thematic saturation within each stakeholder group when no new themes were emerging in later transcripts. The codebook, example code application, and themes were shared with the full study team periodically to obtain their input. Coding was conducted in Dedoose (version 10.0.59; SocioCultural Research Consultants, LLC) [24].

To compare perspectives across the fire service community, we grouped participants into 2 categories: firefighters (career firefighters and union leaders) and leadership (department leaders and national leaders). These 2 categories reflect conceptual similarities within groups (eg, union leaders are themselves firefighters who represent firefighters’ interests), and there were similar results within each group. When describing our findings, we primarily refer to these 2 groups.

None of the coauthors have experience as volunteer or career firefighters, which may have impacted the study and the interpretation of results. The interviewer or moderator participated in ride-alongs with Maryland firefighters to gain insights into firefighting and fire service culture and discussed data privacy issues with local fire service collaborators in Maryland and Virginia to inform the study materials and analyses. One author is a commissioner of a Maryland fire department and may have brought a management perspective to the study. The authors discussed the study findings with study partners in the fire service to help interpret the results. The coders also consulted researchers with extensive experience conducting research with the fire service. This study follows the COREQ (Consolidated Criteria for Reporting Qualitative Research) guideline (Checklist 1) [25].

### Ethical Considerations

This research was approved by the Johns Hopkins Bloomberg School of Public Health Institutional Review Board (24091). We obtained oral consent from all participants. Participants were not compensated for their participation. We deidentified interview transcripts to protect participant privacy.

## Results

### Sample

The sample included a total of 65 participants from 35 interviews and 4 focus groups (5‐9 participants in each): 31 career firefighters, 2 union leaders, 21 department leaders, and 11 national leaders (Table 1).

**Table 1.** Interview and focus group participant roles by jurisdiction^a^.

Participants	Virginia, n	Maryland, n	National, n	Total, n
Firefighters				
Career firefighters	30	1	—^b^	31
Union leaders	1	1	—	2
Leadership				
Department	7	14	—	21
Government	—	—	3	3
Advocacy	—	—	6	6
Research	—	—	2	2
Total	38	16	11	65

a The data were collected from March to November 2023.

b Not applicable.

We encountered data collection challenges in Maryland, which limited the firefighter sample in that state (1 Maryland firefighter participated in a key informant interview compared to 30 Virginia firefighters in focus groups); this limitation is discussed in the *Discussion* section. Overall, our sample was largely White and male, which reflects the fire service’s gender and race demographics [26]. The leadership sample was more diverse in terms of race and gender: 25% (8/32) of leaders were non-White versus 9% (3/33) of firefighters, and 34% (11/32) of the leadership participants were women versus 12% (4/33) of firefighters. Leaders were also older and more experienced than firefighters: the mean age of leadership participants was 47.7 (SD 10.2) years versus 35.9 (SD 7.5) years, and the mean years of experience was 24.6 (SD 11.9) years versus 11.8 (SD 8.8) years. Table 2 contains self-reported demographic characteristics. Focus groups were, on average, 77 minutes. Firefighter interviews averaged 39 minutes, and leadership interviews averaged 45 minutes.

**Table 2.** Interview and focus group participant characteristics by role^a^.

Participant characteristics	Firefighters (n=33)	Leadership (n=32)
Gender, n (%)		
Male	29 (88)	21 (66)
Female	4 (12)	11 (34)
Race, n (%)		
White	30 (91)	24 (75)
Non-White	3 (9)	8 (25)
Age (y), mean (SD)	35.9 (7.5)	47.7 (10.2)
Experience (y), mean (SD)	11.8 (8.8)	24.6 (11.9)

a The table shows self-reported demographic characteristics. One firefighter did not report their age. Two leadership participants did not report their years of experience. The data were collected from March to November 2023.

### Themes

#### Overview

We identified four themes: (1) acceptability of current data access, sharing, and reporting practices; (2) data sharing and access preferences; (3) appropriate use of firefighter data; and (4) need for improved communication about data. Table 3 shows the themes and provides an overview of themes by group (firefighters and leadership).

**Table 3.** Overview of themes^a^.

Themes	Leadership	Firefighters
Acceptability of current data access, sharing, and reporting practices	All participants described limited sharing of identifiable data and adequate protections for most health and safety data.	All participants described limited sharing of identifiable data and adequate protections for most health and safety data.
Data sharing and access preferences	Leaders described concerns about data sharing and ideal conditions for facilitating data collection, such as deidentification and limiting sharing.	Firefighters largely did not share these concerns but favored deidentification and identified sensitive kinds of information, such as mental health data.
Appropriate use of firefighter data	Leaders believed that firefighters were resistant to sharing information based on fear of termination or job impacts; many linked this to fears about losing identity as a firefighter.	For the most part, firefighters did not share these concerns.
Need for improved communication about data	Leaders acknowledged the importance of communicating well about data.	Firefighters and advocates emphasized the need for data interpretation and dissemination.

a The table shows themes from focus groups and interviews with firefighters and fire service leaders (March to November 2023).

#### Acceptability of Current Data Access, Sharing, and Reporting Practices

Within departments, firefighters and leadership described limited sharing of firefighter health and safety data, and most believed that such data were adequately protected by their departments. Nearly all leaders indicated that access to identifiable firefighter health or safety information was granted on a need-to-know basis. For instance, injury information was accessible only to health and safety staff. Most firefighters were required to complete an annual physical exam, but most departments did not receive detailed reports of members’ annual physical results, just a notification of whether they passed or failed.

Externally, department leaders mentioned being legally required to report injuries to government agencies, such as the Occupational Safety and Health Administration, and third parties, such as the National Fire Protection Association and the International Association of Fire Fighters union. Most firefighters were uncertain about how the data might be reported externally but assumed that injury information was shared with government agencies or third parties and viewed this sharing as acceptable.

For the most part, firefighters appeared comfortable with the kinds and amounts of information they shared with their departments, as well as how this information was accessed by department leaders. Some simply did not express strong views on current data sharing practices, whereas others shared that efforts to limit data sharing were sufficient. Others stated that sharing personal information related to their physical health simply came with working in a high-risk, public job.

*I don’t have any real experiences or bad or positive experiences with my data in the department, except for just our health as in our yearly physicals and then stress tests and things like that. And I haven’t had any issues with those or anything.*

[Firefighter]

*I’m sure no one loves to give all their personal information to an employer, but it’s probably necessary in this particular field.*

[Firefighter]

While privacy protocols and confidentiality were reported to be formally upheld, the culture of informal information sharing within departments differed; many firefighters and a few leaders noted that news about injuries traveled quickly.

*Just like with the injury thing, you can decipher who’s, we heard, oh, such and such went to [...] the mental health rehab center. Oh, this guy’s on injury leave for the next three months. Wonder where he is. We can find out things by digging.*

[Firefighter]

Although a few individuals were troubled by how quickly news about health diagnoses or injuries could spread in their departments, they pointed out that this typically occurred through informal channels in which firefighters shared information with each other, not through leadership.

#### Data Sharing and Access Preferences

While most firefighters were comfortable with their departmental data sharing practices, some expressed preferences regarding the kinds of information they were comfortable sharing and with whom they were comfortable sharing it. Many had an ideological preference for limiting access to identifiable information and opting for aggregated data sharing both within and outside of departments. Union representatives concurred—they described encountering broad resistance among firefighters to sharing medical information, although the firefighters in our sample did not express these concerns. Firefighters were generally supportive of sharing information with researchers conducting health and safety research, although some described their frustration that they did not receive individual results or overall results of studies in which they participated (described in more detail below).

Reluctance to share identifiable information appeared to be heightened based on the sensitivity of the data in question. One female firefighter pointed out that she viewed reproductive health information as more private than physical injury information. Mental health data were highly valued by most participants—firefighters emphasized the need for more mental health research and services—but they characterized mental health data as complicated to collect and access. Firefighters generally agreed that mental health data were closely held, both among leadership and peer networks, but participants were still concerned about who might have access to identifiable sensitive information. In one focus group, firefighters described their dismay when asked to complete a mental health research survey that was theoretically anonymous but asked for information that participants believed could be used to identify them. Several firefighters explained:

*Participant 4: Well, but that survey we just did, it has a lot of pretty in-depth. If you take it seriously, it has a lot of in-depth personal mental health [questions]. Like, for example, I think there’s some on there like, have you ever considered committing suicide? Like, how many times in the last week have you felt like, that you were too depressed to come to work? ...*

*Participant 3: And you don’t know where it’s going.*

*Participant 4: You don’t know where it’s going.*

*Many: Yeah.*

*Participant 4: Then you get to the end. It’s like basically you got to describe your eye color, you know.* [laughter]

[Firefighters]

Leadership was aligned with firefighters in preferring that the data be deidentified or aggregated for research and prevention. For some, this was a matter of principle. Others thought that firefighters were less likely to share information if they believed it would be identifiable:

*Even if you tell them it’s going to nobody but me or nobody but this specific office, they are less likely to participate if there is identifying information.*

[Department leader]

Others indicated that firefighters were reluctant to share mental or behavioral health information due to fear of reprisal and the sensitive nature of these data. Some research and advocacy participants thought that firefighters were more likely to share information with external entities than within their departments. There were mixed views among leaders about whether firefighters were opposed to sharing data within departments on principle, with some describing widespread privacy concerns and others indicating that such views were rare.

Beyond individual-level data collection and sharing, a small number of leaders expressed a desire to expand access to population-level data across the fire service. These participants described a tension between using data to advance health and safety priorities while respecting individual privacy rights. Several cited structural challenges in securely storing or accessing data, which prevented this kind of population-level analysis, but they believed it would be useful in the future.

#### Appropriate Use of Firefighter Data

Firefighters in our sample expressed few concerns about how their data were currently being used, whereas leadership described their views on appropriate ways of utilizing firefighter data and recalled instances in which they had heard concerns from firefighters. Leaders emphasized the importance of using firefighter data to improve health and safety but acknowledged that this could raise concerns about data being used against firefighters. Several department leaders stressed that health and safety data should be used in nonpunitive ways:

*We try really hard in our office, at least the safety office, that we are not a punitive type of section of the department. Because once we become punitive, people stop talking to us. People stop trusting us. And our job is to try to make things safer for them. And we can’t make things safer if we don’t really truly understand what the problems are, you know?*

[Department leader]

Leaders across all subgroups, as well as union representatives, believed that firefighters were fearful that sharing health or injury information could result in job loss or changes in job status, ultimately impacting their identities as firefighters. Many described encountering concerns among some firefighters that if they did not pass medical or fitness exams, they would be terminated. Most department leaders believed these views were unfounded and expressed a desire to use these exams for health and safety purposes:

*Because you have firefighters that will be like, “a machine or a doctor or this METS [metabolic equivalents] does not dictate on how I can fight fire,” which is true ... [but] all the research they’ve done with this, says that if your METS are below eight, you are at a higher risk of dying of an MI [myocardial infarction] or something out on fire scene. So that’s the biggest issue I feel in the fire service. Everyone thinks that we’re trying to take their job away or take a ton of money out of their pocket, what they don’t understand is, no, we’re trying to keep you healthy and safe and everything like that.*

[Department leader]

In other cases, leaders anticipated firefighter concerns that sharing health information would adversely affect their day-to-day work even if their employment status remained unchanged:

*I know a couple cases [of] people that never reported anything because they didn’t want to go through the process. Because you got to go to the county clinic and then you got go to the headquarters, and most people get into this job for adrenaline rush and helping people. And then you’re on modified duty, sitting behind a desk with limitations.*

[Department leader]

Several individuals framed these concerns as fears of losing their identity as a firefighter:

T*heir fear isn’t so much losing their job, their fear is losing their identity, who they are.*

[Advocate and department leader]

While these concerns were widespread among leaders, most firefighters did not discuss job impacts. A few were worried that their annual physicals had become so in-depth that they might identify health risks for which they could be terminated or moved to light duty, although this perspective arose in only one focus group. Union leaders attributed hesitation about sharing medical information to underlying concerns about fitness for duty and privacy but were still supportive of requiring annual physical exams.

#### Calls for Improved Communication About Data

As noted earlier, many participants advocated for sharing data with firefighters. However, participants also described shortcomings in how departments or researchers currently communicate with firefighters.

Regardless of how data originated, department and national leaders strongly endorsed the importance of returning individual data and interpretations of data back to firefighters. Many supported sharing information directly with individual firefighters, such as returning each individual’s medical data from an annual exam to empower them to make healthy choices. Participants also emphasized the importance of communicating aggregated and analyzed data to provide education on topics, such as injury prevention or fitness practices. Department leaders and advocates highlighted the importance of partnering with trusted leaders, such as union officials, and communicating effectively about the purpose of data collection. A small number of participants described challenges in sharing data effectively, noting that the fire service or their department did not have a strong track record of effectively communicating about data.

Despite this widespread endorsement of high-quality communication from leadership, many firefighters noted that there was significant room for improvement. Firefighters were vocal about the need for better communication regarding data in general and better interpretation of the data that they provided for departmental or research purposes. Several firefighters wanted more information about the purpose behind data collection efforts or the significance of individual results. For instance, some described not knowing how to interpret annual physical exam results:

*I’m going to voice off. I think the value sucks, to be honest. They [the fire department] do blood work and they do a full physical but nine times out of 10 we get a paper back that says, hey, there was something abnormal about your blood work or something we found, and that’s it. No follow up, no telling you what it actually is. And to actually follow up with them to find out is really hard. They do the work, but they don’t actually give you the results.*

[Firefighter]

Others were frustrated with the lack of research results described earlier. The lack of communication about their own data and study results led them to conclude that a large share of data collection has no purpose or ultimate benefit to firefighters.

*Participant 2: Well look at the urine samples that they [researchers] would collect, stuff like that. They had us give a urine sample after we got back from fires to see, hey, do you have whatever…*

*Participant 9: This has been absorbed into your bloodstream.*

*Participant 2: Arsenic, you know, absorbed into your body.*

*Participant 3: We did it for two years and got…*

*Participant 5: And they lost funding and...*

*Participant 2: Right. They lost funding and it went away. Or every sample you gave, you got zero back from…*

*Participant 5: No idea.*

[Firefighters]

## Discussion

Our study examined firefighters’ and fire service leaders’ data privacy preferences and identified opportunities for fire departments and researchers to ensure firefighter data are appropriately safeguarded and utilized. Leaders had more concerns about data use and data sharing, attributed in part to anticipated firefighter privacy concerns, whereas firefighters held general preferences for deidentifying data and limiting sharing, particularly for sensitive information, such as mental health data. Both groups called for improved data communication.

Interestingly, leaders in our sample voiced more concerns about current data practices than firefighters, and leaders were more attuned to the need for and barriers to ethical data collection, use, and sharing. This might reflect the fact that leadership was more likely to encounter diverse views on data than firefighters working in a single fire station or to hear concerns held by a small number of firefighters but elevated to management. It might also reflect the responsibilities that accompanied their roles—leaders who regularly interacted with firefighter data, or who were responsible for policy implementation, may have had more opportunities to consider the ethical and legal obligations surrounding such data. This aligns with research from our study team examining perceptions of the value of data in the fire service, which found that fire service leaders identified more use cases for occupational health and safety data than firefighters [27]. Leaders were likely more familiar with occupational health and safety data, more likely to value it, and therefore more likely to have strong views on the privacy of that data. Although leaders had more specific concerns about privacy practices than firefighters, both groups identified opportunities to limit access to sensitive data and improve communication.

The launch of new systems, such as NERIS, offers opportunities to adopt a “privacy by design” or “value-sensitive design” approach in which safeguards for privacy or other values are built directly into digital health systems [28-30]. Privacy by design focuses on privacy protections, whereas value-sensitive design accounts for moral values such as privacy, in addition to other values such as ease of use or safety benefits. Value-sensitive design may be a useful framework for fire service technology developers, who may be designing technologies to enhance one moral end (eg, protecting worker health and safety) but need to balance other moral values (eg, protecting privacy) and practical design considerations (eg, usability of a smartphone app for firefighters to track injuries).

For instance, firefighters in our sample expressed hesitancy to share sensitive information about their mental health despite recognizing a need for more mental health research and screening. A value-sensitive mental health data collection system in a mobile app could be designed to anonymize responses, impose limits on collecting sensitive data (eg, suicidal ideation), or place limits on who has access to this information (eg, union leaders instead of department leaders). Leadership identified a broader array of potential data protections, such as limiting identifiable data collection and avoiding punitive use of health and safety data. The developers of digital health interventions for the fire service could take these preferences into account. For example, the developers of data surveillance systems, such as NERIS, could address these priorities by limiting the collection of identifiable information when gathering population-level injury data. Fire service technology companies could design wearable devices and smartphone apps that incorporate privacy features, such as allowing firefighters to opt into sharing specific identifiers or kinds of data with developers, their employers, researchers, or government officials. While these findings have implications for a range of digital health technologies and developers, fire departments, researchers, and government agencies can also help develop policies and practices accompanying these technical safeguards to ensure that health and safety data are used in protective, not punitive, ways.

Our findings also emphasize the importance of transparent and high-quality communication about firefighter health and safety data, both in research and practice settings. Many of the firefighters we spoke with were broadly supportive of research on topics, such as cancer, but were apathetic about their own contributions. Several described their frustration that after sharing sensitive information, such as symptoms of psychological distress or biospecimens, they never heard about the purpose or outcome of a study in which they participated. There are clear and well-established ethical imperatives for communicating the purpose of a study to research participants [31]. Our findings suggest there is strong demand from firefighters for researchers to clearly communicate about the objectives and conclusions of their work. Understanding these kinds of communication best practices will be particularly important as the National Institute for Occupational Safety and Health rolls out the National Firefighter Registry, a national-level longitudinal research effort. There are fewer well-established norms for communication about this kind of public health surveillance as opposed to individual research studies. Additional research on this topic could help establish guidelines on if and how firefighters want to receive information from surveillance activities such as the National Firefighter Registry.

Communicating individual research results is a more complicated endeavor; there is robust debate about when it is appropriate to return individual research results to participants, based on the kinds of data in question and how results might be communicated [32,33]. However, there is growing consensus that study participants want to receive medically significant and actionable findings, which is consistent with the views of firefighters in our sample [32,34]. While researchers cannot always ensure that findings are translated into practice, they can commit to providing research participants with individual results that might increase awareness about their own health and to sharing the conclusions of a study. Research ethics literature offers best practices for how researchers can communicate with participants, including offering context, using clear and simple language, and providing opportunities for questions [35,36].

Our findings also reveal the need for more research on the best manner in which to return health or medical information collected in occupational settings (not for research), for instance, from an annual physical exam or a department-led safety initiative. Researchers and employers operate in different environments, and the nature of data collection will vary accordingly. For example, firefighters may be required to participate in an annual exam as part of the terms of their employment, whereas research participants voluntarily enroll in a study. Research studies aim to produce generalizable knowledge and societal benefits, whereas a department’s safety efforts might have limited population-level benefits. More work is needed to determine the ethical obligations of employers in communicating health and safety data to their employees. Ensuring ethical, high-quality communication about these kinds of data may not only benefit individual firefighters but could also help bolster trust in data collection efforts within the fire service writ large.

Finally, our findings reflect a need for robust ethical guidance on how to appropriately handle health and safety data in occupational settings, including the establishment of ethical principles as well as detailed, practical guidance on privacy topics. Although our participants did not raise major alarms about unethical data practices, they described legitimate concerns about ensuring firefighter data privacy while calling for large amounts of new data collection. The concerns that health and safety data could be used in punitive ways—to fire someone or change their job responsibilities without cause—reflect serious potential repercussions from insufficient data privacy protections. A few experts have discussed the need for confidential data practices for health and safety data, but guidelines for ethical data practices in any occupational setting remain general [37]. The COVID-19 pandemic revealed the need for ethics and data governance guidance for employers, as they began to deploy biometric screenings and digital contact tracing apps to attempt to control disease spread in workplaces, often with little ethical guidance [38,39]. While there were efforts to develop guidelines specific to COVID-19 [40], more work is needed to establish guidelines for the routine collection of occupational health and safety data, outside of a public health emergency. The continued refinement of ethical frameworks specific to occupational health and safety, as well as practical guidance on how to implement these frameworks, will help employers implement new data efforts in an ethical manner.

Our results should be interpreted with several key limitations in mind. Our findings reflect the specific geographic regions and fire departments in our sample. Fire departments were recruited from study team networks and had previously participated in research, which may have influenced their views toward research and data privacy. We encountered recruitment challenges in Maryland that limited the size of our firefighter sample in that state. We found similar results across Maryland and Virginia, but a larger sample may have captured additional viewpoints. Attitudes toward data privacy may differ based on department culture, trust in leadership, staffing (eg, combination, volunteer, or career), and geography. Future research should explore this topic in a wider range of departments. Our sample was also largely White and male. While this reflects the overall demographics of the fire service [26], evidence from other settings suggests that women and racial minorities experience data and technology in distinct and often inequitable ways and thus may have unique privacy preferences [41-44]. More research is needed to understand how perspectives on occupational health and safety data privacy might vary based on gender and race.

Our findings offer insights into pathways for enhancing firefighter data privacy. Incorporating firefighters’ privacy preferences into digital health data systems, for instance, by deidentifying firefighter data and limiting data access, may build confidence and trust in data collection efforts. Improving communication about data will address firefighters’ desire to hear more about the purpose and impact of data collection. By addressing firefighters’ privacy concerns and conducting high-quality, tailored communication, government, research, and department-level leaders can foster trust in data collection efforts and ensure that firefighter data are used in ethical and acceptable ways to protect their health and safety.

## Supplementary material

10.2196/84465 Multimedia Appendix 1 Interview and focus group discussion topics.

10.2196/84465 Checklist 1 COREQ checklist.

## Abbreviations

COREQ: Consolidated Criteria for Reporting Qualitative Research
NERIS: National Emergency Response Information System
