# Supplementary material for: Ethical Handling of Occupational Health and Safety Data in the Fire Service: Empirical Interview and Focus Group Study of Firefighter and Fire Service Leadership Privacy Preferences
Source: J Med Internet Res. 2026 Apr 2;28:e84465. doi: 10.2196/84465 (PMC13045656; doi:10.2196/84465)
Supplement: Multimedia Appendix 1 [file jmir-v28-e84465-s001.docx]

**Appendix 1. Focus group discussion topics**

| **Current data practices** | | |
| --- | --- | --- |
|  | Data collection | - Are there things that you don’t want to share with management? What about the union? Why? |
|  | Data use | - What is health and safety information used for? [compliance, research, safety, workers comp, human resources] |
|  | Data sharing | - Who do you think this information is shared with in the department? - What about outside of the department? [government, research, contractors] - What do you think about the level of information you share with the department right now? |

Note: Discussion topics and questions are examples of initial questions. The moderator asked follow up questions to probe within each of these topics.

**Appendix 2. Focus group exit survey**

Participant number:

1. What is your age?
2. Do you identify as:
   1. Female
   2. Male
   3. Nonbinary/other
3. Do you identify as (select all that apply):
   1. Asian
   2. Black
   3. White
   4. Hispanic or Latino/Latina
   5. Native American or Alaska Native
   6. Other
4. Do you use a fitness tracker (e.g. a FitBit)?
   1. Yes
   2. No
5. Do you use a smartwatch (e.g. an Apple watch)?
   1. Yes
   2. No
6. Do you use social media platforms (e.g. Twitter, Facebook, or Instagram)?
   1. Yes
   2. No
   3. Not sure (please elaborate):
7. Do you document your exposures using an app like NFORS or through your own recordkeeping?
   1. Yes – I use an app like NFORS
   2. Yes – I use my own recordkeeping system
   3. No

**Appendix 3. Interview topics**

Before we begin our discussion, I want to ask you a few demographic and background questions:

1. What is your age?
2. What is your gender?
3. What is your race/ethnicity?
4. What is your rank?
5. How many years of experience do you have in the fire service?
6. Do you use a fitness tracker (e.g. a FitBit)?
7. Do you use a smartwatch (e.g. an Apple watch)?
8. Do you use social media platforms (e.g. Twitter, Facebook, or Instagram)?

| **Current data practices** | | |
| --- | --- | --- |
|  | Data collection | - Are there types of information you would like to have but don’t currently have access to? |
|  | Data use | - How would you say the department/your organization currently uses safety-related data (e.g. injury data)? |
|  | Data sharing | - Who is that data shared with in the department/organization? What about outside of the department/organization? - Do you have partnerships with any professional societies or researchers? |

Note: Interview topics and questions are examples of initial questions. The interviewer asked follow up questions to probe within each of these topics.
